# Supplementary material for: Acute Oxidative Stress Can Paradoxically Suppress Human NRF2 Protein Synthesis by Inhibiting Global Protein Translation
Source: Antioxidants (Basel). 2023 Sep 7;12(9):1735. doi: 10.3390/antiox12091735 (PMC10525356; doi:10.3390/antiox12091735)
Supplement: Supplementary file 1 [file antioxidants-12-01735-s001.zip › antioxidants-2518139-supplementary.pdf]

Supplementary Figures

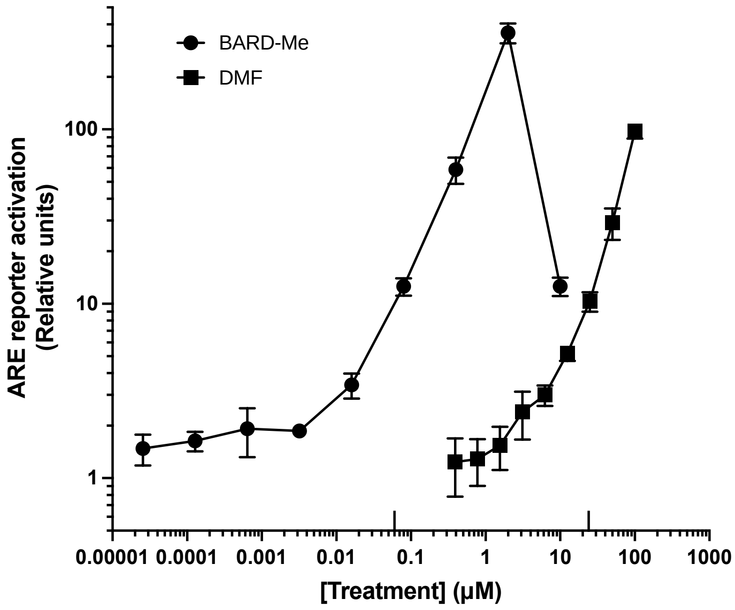

Supplementary Figure S1. The NRF2-activating electrophiles bardoxolone methyl (BARD-Me) and dimethyl fumarate (DMF) induce the ARE reporter by ≥100-fold. Cells transfected with the ARE luciferase reporter and a control reporter were treated for 18 h at the concentrations shown. Luciferase reporter activity in lysates is ARE reporter activity divided by control reporter activity, $n = 6$, then normalized to vehicle-treated cells. Where error bars are not shown, they are shorter than the height of the symbol.

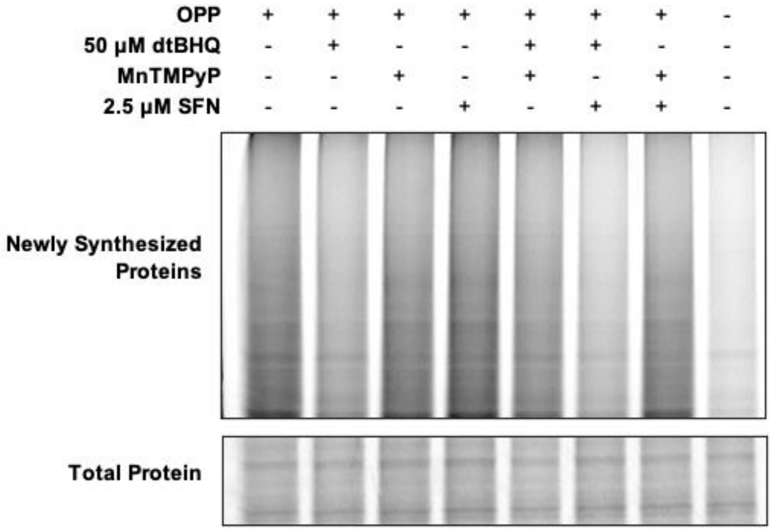

Supplementary Figure S2. The ROS-generating molecule dtBHQ, but not the electrophilic NRF2 activator sulforaphane, inhibits global protein synthesis. Cells were treated as indicated for 2 h prior to harvest and global protein synthesis analysis. Total protein analysis was performed by Coommassie staining. This image is representative of two biological replicates with two technical replicates each. Quantitation of these is shown in Figure 4D.
